# Implementation of an integrated care programme to avoid fragility fractures of the hip in older adults in 18 Bavarian hospitals – study protocol for the cluster-randomised controlled fracture liaison service FLS-CARE

**DOI:** 10.1186/s12877-020-01966-1

**Published:** 2021-01-12

**Authors:** Isabel Geiger, Christian Kammerlander, Christine Höfer, Ruth Volland, Jörg Trinemeier, Martina Henschelchen, Thomas Friess, FLS-CARE study group, Ursula Andrae, Christian Stäudel, Hans-Joachim Andress, Theresa Liese, Maik Behnke, Milos Stojanovic, Ulrich Brunner, Sabine Schreiber, Georg Gradl, Benedikt Fürst, Stefan Grote, Constantin Schmid, Sebastian Grüninger, Susanne Wicklein, Tobias Lewens, Kolja Gelse, Thomas Löffler, Martin Bendiks, Rainer H. Meffert, Benedikt Schmitz, Carl Neuerburg, Martin Odenwald, Maximilian Thoma, Paul Schmitz, Rainer Kretschmer, Rupert Schupfner, Eva Pfeifer, Igor Solovyov, Christian Linder, Ulla Stumpf, Jutta Werther, Annabel Fenwick, Christian Zeckey, Joseph Ditto, Wolfgang Böcker, Leonie Sundmacher

**Affiliations:** 1 Department of Health Services Management at LMU (LMU-HSM), Munich, Germany; 2 Department of General, Trauma and Reconstructive Surgery, Hospital of the Ludwig-Maximilians-University (LMU) Munich, Munich, Germany; 3 AUC – Academy for Trauma Surgery GmbH, Munich, Cologne, Germany; 4 PVM Versorgungsmanagement GmbH, Speyer, Germany; 5 München Klinik Schwabing, Munich, Germany; 6 Helios Klinik München Perlach, Munich, Germany; 7 REGIOMED Klinikum Coburg GmbH, Coburg, Germany; 8 Krankenhaus Agatharied GmbH, Hausham, Germany; 9 München Klinik Harlaching, Munich, Germany; 10 Klinikum St. Elisabeth Straubing GmbH, Straubing, Germany; 11 Klinikum Nürnberg Süd, Nuremberg, Germany; 12 Klinikum Traunstein, Traunstein, Germany; 13 Klinik Weilheim, Weilheim, Germany; 14 Universitätsklinik Würzburg, Wurzburg, Germany; 15 LMU Klinikum Großhadern, Munich, Germany; 16 München Klinik Bogenhausen, Munich, Germany; 17 Caritas Hospital St. Josef, Regensburg, Germany; 18 Klinikum Bayreuth, Bayreuth, Germany; 19 Klinik Schongau, Schongau, Germany; 20 Uniklinikum Augsburg, Augsburg, Germany; 21 Romed Klinik Rosenheim, Rosenheim, Germany; 22 Chair of Health Economics, Technical University of Munich, Munich, Germany

**Keywords:** Osteoporosis, Fragility fracture, Fracture liaison service, Hip fracture, Integrated care, Secondary prevention

## Abstract

**Background:**

The economic and public health burden of fragility fractures of the hip in Germany is high. The likelihood of requiring long-term care and the risk of suffering from a secondary fracture increases substantially after sustaining an initial fracture. Neither appropriate confirmatory diagnostics of the suspected underlying osteoporosis nor therapy, which are well-recognised approaches to reduce the burden of fragility fractures, are routinely initiated in the German healthcare system. Therefore, the aim of the study FLS-CARE is to evaluate whether a coordinated care programme can close the prevention gap for patients suffering from a fragility hip fracture through the implementation of systematic diagnostics, a falls prevention programme and guideline-adherent interventions based on the Fracture Liaison Services model.

**Methods:**

The study is set up as a non-blinded, cluster-randomised, controlled trial with unequal cluster sizes. Allocation to intervention group (FLS-CARE) and control group (usual care) follows an allocation ratio of 1:1 using trauma centres as the unit of allocation. Sample size calculations resulted in a total of 1216 patients (608 patients per group distributed over 9 clusters) needed for the analysis. After informed consent, all participants are assessed directly at discharge, after 3 months, 12 months and 24 months. The primary outcome measure of the study is the secondary fracture rate 24 months after initial hip fracture. Secondary outcomes include differences in the number of falls, mortality, quality-adjusted life years, activities of daily living and mobility.

**Discussion:**

This study is the first to assess the effectiveness and cost-effectiveness/utility of FLS implementation in Germany. Findings of the process evaluation will also shed light on potential barriers to the implementation of FLS in the context of the German healthcare system. Challenges for the study include the successful integration of the outpatient sector as well as the future course of the coronavirus pandemic in 2020 and its influence on the intervention.

**Trial registration:**

German Clinical Trial Register (DRKS) 00022237, prospectively registered 2020-07-09

## Background

With increasing life expectancy in Germany, the number of people with age-associated progressive metabolic bone disease, so-called osteoporosis, rises steadily as well. Suffering from osteoporosis poses a tremendous risk for those affected to sustain bone fractures as a result of lowered bone density. According to the prevalence extrapolations of Hadji et al. (2013) [1], 6.3 million people in Germany suffer from osteoporosis and 885,000 new cases are expected to occur annually. Women aged 50 years or above are particularly prone to develop osteoporosis and, therefore, have a significantly higher lifetime risk of sustaining a major osteoporotic fracture (i.e. arm, hip and spine) compared with men [2].

In Germany, osteoporosis-related healthcare costs amounted to 4.5 billion Euros in 2009 [3]. Almost a decade later, osteoporosis-associated fractures were responsible for annual expenses of more than 11 billion Euros. Between 2017 and 2030, osteoporosis-induced healthcare costs are expected to increase further up to 14 billion Euros per year. Even though hip fractures represent a fifth of all fragility fractures, they make up half of the total fracture-related healthcare costs [2].

Fractures of the hip are considered to be the most studied osteoporosis-related bone fractures because of not only their high associated costs but also their great impact on patients’ survival rate and quality of life. For example, the likelihood of requiring long-term care is estimated to be four-fold after sustaining an osteoporosis-related hip fracture [4, 5]. Moreover, the overall risk of an individual suffering from any secondary fracture increases substantially after an initial injury [2].

The most likely cause of secondary fractures lies in underlying, untreated osteoporosis. Thus, to reduce the economic and public health burden of fragility fractures, appropriate diagnostic testing (e.g. bone mineral density) to confirm whether osteoporosis is the underlying root cause is essential in order to subsequently initiate adequate treatment to prevent future fragility fractures. The International Osteoporosis Foundation (IOF) [2], however, reported that neither appropriate diagnostics of the suspected underlying osteoporosis nor therapy such as measures of fall prevention and/or drug treatment are routinely initiated in German hospitals or, afterwards, in the ambulatory care sector. In fact, only 40% of all patients suffering from a fragility fracture in the high-risk group (women over 50 years) were found to receive appropriate diagnostics, leaving 60% of the patients unrecognised [6]. In addition, drug persistency of those women receiving treatment over a time frame of 2 years was found to be reduced to 40% or lower [7]. In 2018, only 20% of hip fracture patients (> 70 years, low-energy trauma) treated in German hospitals with certified orthogeriatric co-management (AltersTraumaZentrum DGU®) reported pre-fracture osteoporosis therapy; of these, 22% received specific medication [8].

International studies have shown that coordinated care models specifically designed for secondary fracture prevention, so-called Fracture Liaison Services (FLS), can lead to a substantial increase in appropriate assessment of the root cause and guideline-adherent therapy of fragility fractures, thus significantly reducing secondary fractures [9]. FLS have also been reported to be a cost-effective treatment option for hip fractures in Canada, Japan, the US and the UK. However, the FLS intervention varied substantially between countries [4].

## Objectives

FLS has not been implemented in German hospitals as a standard of care so far. However, because of the great burden of osteoporotic hip fractures combined with low preventive treatments, patients may benefit greatly from its implementation. Therefore, the overall objective of the study FLS-CARE is to test whether the coordinated care programme can close the care gap for patients suffering from a fragility hip fracture through implementation of systematic diagnostics and guideline-adherent interventions based on the FLS model.

The primary hypothesis (H_1_) is that, under the given circumstances of the German healthcare system, the implementation of FLS-CARE leads to a significant reduction in secondary fracture rates after initial hip fracture within a time frame of 2 years. Secondary hypotheses include that the implementation of FLS-CARE prevents falls (H_2_), lowers mortality (H_3_), increases quality of life (H_4_), patient satisfaction (H_5_) and therapy adherence (H_6_) and is a cost-effective intervention (H_7_).

## Methods

### Trial design

The study is set up as a non-blinded, cluster-randomised, controlled trial with unequal cluster sizes. Allocation to intervention group (FLS-CARE) and control group (usual care) follows an allocation ratio of 1:1 using hospitals (trauma centres) as the unit of allocation. In total, 18 study sites (9 hospitals per group) across Bavaria participated in the study. The cluster-randomised study design was chosen to avoid contamination bias by clinical staff and other patients within one centre.

For the purpose of this study, only trauma centres located in one federal state of Germany (Bavaria) are included.

### Study population and eligibility criteria

**Fig. 1:**
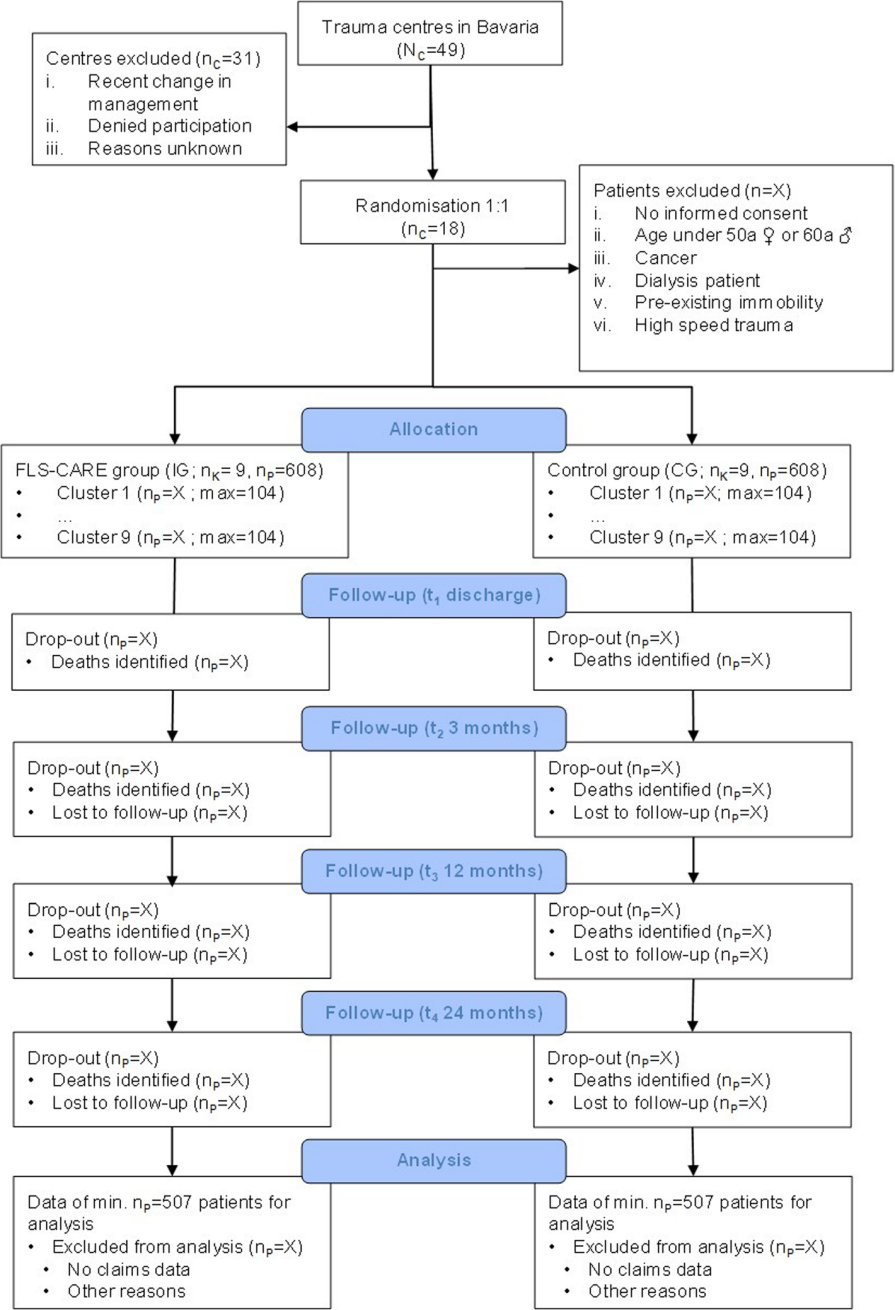
Flowchart of the FLS-CARE study. N_C_ refers to the overall number of trauma centres; n_P_ refers to the number of patients

Out of 49 potentially suitable hospitals in Bavaria, 18 centres were recruited for the study through a letter of invitation. Hospitals were excluded from participation if a recent change in management occurred that could influence implementation of the study, if they failed to provide a letter of intent or refused to participate in the study (see Fig. 1).

The eligibility criteria for patients to participate in the study are as follows:

#### Inclusion criteria for patients

- Insured by one of six participating sickness funds of the statutory health insurance (SHI), which are estimated to cover approximately 90% of the publicly insured Bavarian population (AOK Bayern, BARMER, DAK-Gesundheit, IKK Classic, BKK Mobil-Oil and Techniker Krankenkasse)
- Hip fracture near the joint (trochanteric fracture (AO type 31 A1–A3) or femoral neck fracture in the context of low-energy trauma (AO type 31 B1–B3, not C1–C3))
- Age ≥ 50 years for women and ≥ 60 years for men
- Written informed consent

#### Exclusion criteria for patients

- Any cancer diagnosis
- Dialysis patients
- Pre-existing immobility
- Currently receiving specific osteoporosis therapy
- High-energy trauma

#### Identification of eligible patients

Depending on the study arm, either FLS-CARE or study nurses identify potential patients using pre-agreed protocols through the hospital information system (HIS). If the screening of eligible patients is successful, the nurse informs the attending physician who is responsible for obtaining informed consent. After receiving details of study participation, every patient has at least 24 h for consideration. Once enrolled in the study, the participant can withdraw within 2 weeks through their sickness funds without giving reasons and thereafter through a declaration of will [10]. Clear guidelines on the withdrawal process are provided for all participants at the time of enrolment.

### Intervention

The new form of healthcare delivery (FLS-CARE) is compared with usual care to assess both effectiveness and cost-effectiveness/utility of the intervention.

#### Intervention group

The FLS-CARE study intends to reduce the frequency of secondary fractures by targeting underlying osteoporosis as the root cause. The study programme includes a guideline-based care concept (FLS) in a network of multiple professionals (physicians, nurses and physiotherapists) from the inpatient and outpatient sectors. Specially trained FLS-CARE nurses play a central role in FLS-CARE as liaison partners for the sectors. They are responsible for guidance between hospital and ambulatory care and health literacy of the patients. They monitor patients’ adherence and ensure the conduct of intervention measures. The FLS-CARE nurses at the study centres, the participating GPs and medical specialists are educated on the principles of FLS and parameter collection for the study.

The FLS model for the patients of the FLS-CARE (intervention) group comprises four main modules:

1. *Diagnostics*: All patients receive an evidence-based diagnostic assessment based on the DVO guidelines [11] and the best practice framework of the IOF [12] during their hospital stay.
2. *Education*: The FLS-CARE nurses highlight the importance of, for example, fall prevention and other physiotherapeutic exercises and motivate patients to follow up their goals for therapy to prevent secondary fractures and to enhance health literacy and behaviour. After discharge, the FLS-CARE nurses make domiciliary visits to assess and educate patients on individual fall risks. They also highlight the nearest support groups for patients suffering from osteoporosis as additional resources.
3. *Therapy*: All patients receive medical and physical therapy as indicated, again in accordance with DVO and IOF guidelines. Treatment plans and adherence are regularly monitored by the FLS-CARE nurses.
4. *Coordination*: The FLS-CARE nurses help to coordinate physical therapy sessions and follow-up appointments (3, 12 and 24 months post fracture) in the outpatient sector at certified GPs and/or medical specialists to ensure the continuity of specific care. In regular telephone calls, FLS-CARE nurses try to improve long-term adherence to therapy.

#### Control group

Participants in the control group will receive treatment as usual with follow-up appointments at identical intervals to the FLS-CARE group. Trained study nurses are responsible for contacting the patients and collecting study parameters from the control group to avoid potential biases.

All trauma centres treating the control group are prohibited from implementing any form of FLS during the study period to avoid potential bias. However, they are not forbidden to practise modules of FLS if they have already been implemented as a standard of care.

### Outcomes

The primary outcome measure of the study is the secondary fracture rate 24 months after initial hip fracture to see whether FLS-CARE serves as an effective tool for secondary prevention under the circumstances of the German healthcare system.

In addition, secondary outcomes, process indicators, health economic parameters and risk factors are collected and evaluated (see Table 1). Secondary outcomes include the absolute and relative number of falls after discharge, the mortality of patients (time to event), changes from baseline in quality-adjusted life years (QALY), activities of daily living (Barthel Index) and mobility (Parker Mobility Score). QALYs are calculated using the standardised EQ-5D-5L questionnaire from the EuroQol Group [13]. Satisfaction with the treatment of the fragility fracture is assessed from all participants after discharge and 24 months after initial fracture using a translated form of the standardised Short Assessment of Patient Satisfaction (SAPS) [14].

**Table 1:**
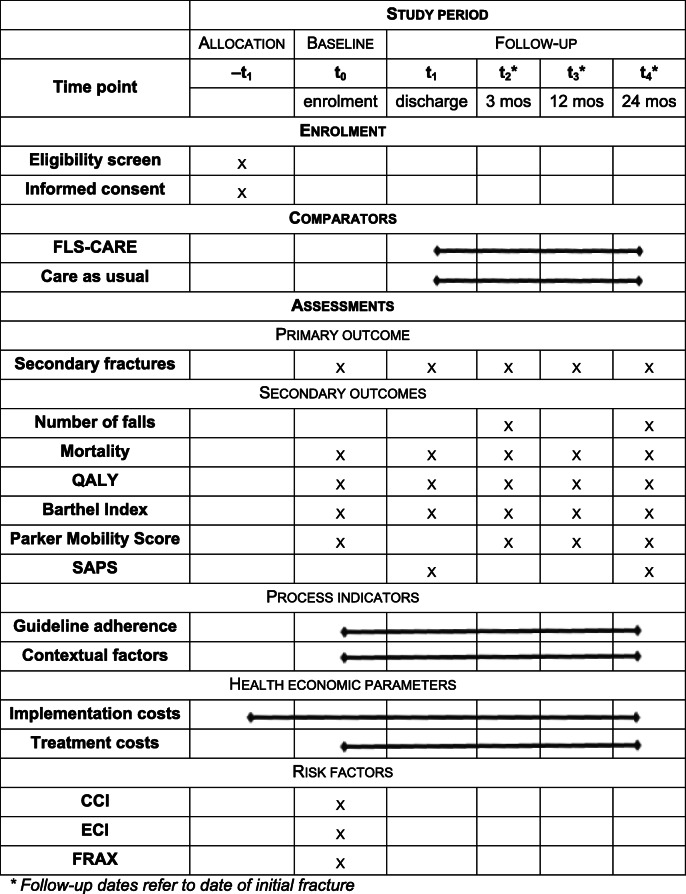
Schedule of enrolment, intervention and assessments

* Follow-up dates refer to date of initial fracture

Process indicators relate to the medical and diagnostic guideline adherence of all participants and are derived from the documentation of FLS-CARE/study nurses as well as insurance claims data from the participating sickness funds. Additionally, contextual factors that facilitate or hinder the intervention and mechanisms of impact are identified through semi-structured interviews and questionnaires with relevant stakeholders in accordance with the Medical Research Council (MRC) guidance on process evaluation of complex interventions [15]. Parameters for the economic evaluation of the health care programme, next to the secondary fracture rate (effectiveness measure) and QALYs (utility measure), include implementation costs of FLS-CARE and overall treatment costs associated with the initial hip fracture from sickness fund data. Risk factors of secondary fractures and potential confounders of the treatment effect are collected using the Charlson Comorbidity Index (CCI), Elixhauser Comorbidity Index (ECI) and the fracture risk assessment tool of the IOF (FRAX®).

### Sample size

Sample size calculation is based on the comparison between the primary endpoint of the FLS-CARE group versus the control group in patients with femoral fragility fractures. Johansson et al. (2017) investigated the follow-up rate in severe osteoporotic fractures in a large study and found that the secondary fracture rate was 26% [16]. However, the exact follow-up period in which the secondary fractures occurred was unclear. In a study by Johnell et al. (2004) [17], a secondary fracture rate of 10% was observed in patients (mean age 80 years) with hip fractures 2 years after the initial fracture. Thus, this rate was assumed to approximate the value for the present control group. Following the method of Hemming et al. (2011) [18] for cluster-randomised studies with unequal cluster sizes, the number of cases per group for a given number of clusters can be determined as follows:

$$ {n}_C=\frac{n_ik\left[1-\rho \right]}{\left[k-{n}_i\left({cv}^2+1\right)\rho \right]}. $$

where *n*_*i*_ corresponds to the individually randomised sample size, *k* to the number of clusters per group, *cv* to the coefficient of variation of a cluster and *ρ* to the intra-cluster correlation.

The participating hospitals are considered as cluster units. For the calculation of the number of patients needed for the analysis, 9 hospitals per group, an intra-cluster correlation of 1% and a coefficient of variation of clusters of 1.37 were assumed. Based on these values, 507 patients are needed per group to find a significant difference of 7% in the secondary fracture rate 2 years after the initial fracture (FLS-CARE group = 3% and control group = 10%) with a power of 80% and a significance level of 5%. The sample size was calculated using GPower 3.1 and R 3.3.2.

In the FAITH study (2014) [19], two surgical methods and their outcome after 2 years were studied in patients with fragility hip fractures. For sample size calculations, a mortality rate of 10% after 2 years and a lost to follow-up rate of 5% were taken into account. Owing to comparable patient populations regarding age and injuries, a similar drop-out rate is assumed for the FLS-CARE study. However, it is expected that the lost to follow-up rate will be 10%, especially in the control group. These considerations result in a total drop-out rate of 20% and a total number of 1216 patients (608 patients per group distributed over 9 clusters).

### Recruitment

FLS-CARE or study nurses are responsible for recruitment to minimise selection bias. Once a patient is admitted to a participating hospital, the nurses are notified through the HIS or following a standardised communication protocol including the nurses and other hospital staff. If initial screening is positive, the attending physician will be informed about the potential study participant. Subsequently, information about either FLS-CARE or potential participation in a control group according to the study site is provided during the hospital stay. If the patient signs the informed consent, he or she, regardless of whether they are assigned to the control or FLS-CARE group, will be registered as a case in the FLS-CARE documentation module.

Duration of recruitment is limited to 12 months and is carried out under regular monitoring (weekly). In order to avoid a disproportionate distribution in one or more clinics, an upper limit of 104 patients per cluster is intended, which corresponds to an average recruitment frequency of two participants per week. Owing to the ongoing COVID-19 pandemic in 2020, the upper limit might be subject to minor adaptation if deemed necessary.

Consent is asked for further scientific use of the clinical data collected during hospital stay and follow-up phase (pseudonymised). Approval is sought to integrate close relatives or caretakers in the communication with the patient following legal regulations.

### Allocation

The allocation ratio of participating hospitals is set to 1:1 with unequal cluster sizes. In order to achieve similar clusters between the FLS-CARE and control groups, the hospitals are stratified by type (local/regional and superregional trauma centre) and location according to a simplified classification (metropolis yes/no). To eliminate selection bias, the trust centre of the evaluating institution of the study (LMU-HSM) was included in the sequence generation process to pseudonymise (conceal) all potential trauma centres before randomisation into FLS-CARE and control groups. The randomisation was performed by LMU-HSM using R 3.3.2.

Owing to the nature of the study, blinding of neither the study participants nor the FLS-CARE/study nurses is feasible.

### Data collection

All participants are interviewed at five points in time (see Table 1 and Fig. 1): directly after informed consent (t_0_), at discharge (t_1_), after 3 months (t_2_), 12 months (t_3_) and 24 months (t_4_) post fracture. For the FLS-CARE group, outcomes are collected by either outpatient physician or FLS-CARE nurses at the follow-up appointments (t_1–4_). Both outpatient physician and FLS-CARE nurse receive prior training in data collection from the FLS-CARE study group. For the control group, study nurses are responsible for data collection.

All follow-up appointments and outcomes are recorded in the specifically developed and highly standardised FLS-CARE tailored IT-based documentation, so-called FLS-CARE documentation modules, to promote data quality.

The FLS-CARE/study nurses also have access to diagnostic, therapy and process data through the aforementioned FLS-CARE documentation modules. They can monitor whether participants comply with follow-up schedules. If a patient does not appear at an appointment, the FLS-CARE/study nurse receives notification and can contact him or her by telephone. If he or she cannot or does not want to attend the follow-up appointment due, the FLS-CARE/study nurse tries to collect the study parameters by telephone.

Participation is not specifically promoted in the intervention group, other than mentioned above, in order to create results that are directly transferable to clinical routine. Missing data and related information are recorded as such in the FLS-CARE documentation modules.

### Data management

Acquisition and documentation of all study data are fully IT based. FLS-CARE uses a SAP Business By Design© platform on a cloud-based IT architecture in combination with clinical trial software provided by the software partners Arvato Systems and ID. This platform solution allows multicentric secure VPN online access for all participating study centres. Databases are run in level 2 certified data centres in Germany. Access to the FLS-CARE application follows strict data security levels for both study centres and the study team. All study forms follow the time points of the participant timeline (see Table 1) using context-specific documentation sheets for both the different time points of data collection and the different user groups. A wide range of software-based plausibility mechanisms is implemented into the documentation forms to define data values and allowed varieties of data entry for every single data field.

#### Confidentiality

Compliance with the legal provisions on data protection and data security within the framework of the FLS-CARE study will be guaranteed by the study group. The collection and processing of data is carried out under strict observance of the legal regulations, in particular the provisions of data protection, medical confidentiality and social secrecy. Decisive are sections 284 et seq*. Fünftes Sozialgesetzbuch* (SGB V) and §§ 67 and 67a *Zehntes Sozialgesetzbuch* (SGB X) on data collection, § 203 *Strafgesetzbuch* (StGB) in conjunction with § 9 *Muster-Berufsordnung für Ärzte* (MBO) on medical confidentiality, § 35 *Erstes Sozialgesetzbuch* (SGB I) on social secrecy and § 5 *Bundesdatenschutzgesetz* (BDSG) on data secrecy.

The data are only forwarded to GPs and medical specialists involved in the study or if the legislator allows the forwarding under defined conditions. The scientific and statistical evaluation of the data collected within the framework of the FLS-CARE study is carried out exclusively with pseudonymised data that preclude any inferences being drawn about the patients.

Data storage takes place in external, certified data centres in Germany. Access to the stored data is only possible by or on behalf of authorised persons.

### Statistical methods

Evaluation of the study will be performed as intention to treat analysis with patients as the unit of observation.

Although the stratification of the cluster aims at evenly distributed patient characteristics, it cannot guarantee perfect comparability of the two groups. Thus, the need for an adjusted model within the regression models is examined at the beginning of the analyses (e.g. due to unequal distribution of relevant baseline characteristics and risk factors). Mixed models are expected to be best fitting for analysing the secondary fracture rate over the study period of 2 years (primary outcome).

Secondary outcomes are descriptively summarised and, if appropriate, analysed within adjusted regression models depending on the type of variable, also considering potential cluster effects. Differences in mortality rates of the FLS-CARE and control group are estimated through survival analyses.

#### Economic evaluation

An economic evaluation of the healthcare programme will establish the cost-effectiveness und cost–utility of FLS-CARE in comparison to standard care.

The results are illustrated as incremental cost-effectiveness and cost–utility ratios, which represent the additional costs incurred in relation to the additional effectiveness or the utility values of FLS-CARE compared with usual care.

Treatment costs are calculated based on sickness fund data from the participating funds. The perspective of the sickness fund is adopted in order to provide exhaustive information on the decision for reimbursement of FLS-CARE for SHI insured patients. The statistical uncertainty is estimated using cost-effectiveness acceptability curves (CEAC).

#### Process evaluation

The statistical analysis and the economic evaluation will be complemented by a process evaluation in accordance with MRC guidance [15] in order to identify contextual factors (facilitators and/or barriers) that could influence the outcomes of the intervention.

For the process evaluation, insights at the macro, meso and micro level will be gained and analysed using mixed methods. This is accomplished through structured questionnaires, evaluation of patient data and semi-structured interviews. A sample of all relevant stakeholders (FLS/study nurses, representatives of hospitals, outpatient physicians and patients) is used to describe which patient-specific (micro), organisational (meso) or systemic (macro) aspects of the implementation should be addressed for transferring FLS-CARE to usual care.

#### Additional analyses

Explorative sensitivity analyses are planned to clarify the dependence of the results on selected parameters (e.g. type of fracture).

The analyses are carried out as intention to treat analysis. Therefore, all patients, even if they are not compliant with the study protocol, will be analysed in their initially allocated group (FLS or control group).

Incomplete datasets are completed, if possible, by multiple imputations using chained equations.

### Oversight and monitoring

The primary investigator is obliged to report the study progress to the health department of the *German Aerospace Centre* (DLR) on a quarterly basis. A more detailed financial and technical report is submitted to the DLR annually. The DLR serves as the executing organisation of the funding institution (GBA). No additional data monitoring committee is established for the purpose of the study.

Any modification of study protocol, milestones and timetables is to be reported to the DLR and, if applicable, to the trial registry. If the adaptations relate to data security in ways that are not covered in the written consent form, all trial participants will be informed in writing. The corresponding ethics committee and the regulatory authorities of the public health insurances will be notified if major changes occur in eligibility criteria and data usage/security.

#### Adverse event reporting

The intervention is based on peer-reviewed guidelines for the treatment of fragility fractures. Therefore, no adverse events are expected to occur through the implementation of FLS-CARE. Nevertheless, the study will be accompanied by a process evaluation based on recommendations of the MRC to detect any unintended pathways and their consequences among other mechanisms of impact at every stage of the intervention [15].

A separate risk assessment for the coronavirus (COVID-19) pandemic in 2020 will be performed by the primary investigators to ensure safety of the participants during the pandemic.

## Discussion

The economic and public health burden of osteoporosis in Germany is high. This study is the first to assess the effectiveness and cost-effectiveness/utility of the implementation of a specifically designed fracture liaison service, called FLS-CARE, to close the prevention gap for patients suffering from a fragility hip fracture in Germany. Through targeting low-energy trauma hip fractures only, we aim to include individuals who can benefit most from FLS-CARE due to the high likelihood of having undiagnosed osteoporosis as an underlying disease. Thus, the findings of the study will provide insights into whether and how FLS-CARE can reduce secondary fractures. Moreover, findings of the process evaluation will also shed light on potential barriers to the implementation of FLS in the context of the German healthcare system. If FLS-CARE proves to be a successful healthcare service, our study results can additionally support regionwide or nationwide implementation of FLS-CARE.

Germany, like many other European countries, faces the challenge of fragmented care. Thus, one important success factor of the study will be the creation of a multidisciplinary network to ensure the continuity of care. Specifically, the involvement of GPs and medical specialists in the outpatient sector is of great importance. If cooperation between the FLS nurses and the outpatient sector fails, the effectiveness of the intervention may suffer substantially. To counteract this, FLS-CARE has already been introduced to several physician associations to increase awareness of the study and foster the physicians’ willingness to participate in the study.

Another tremendous risk is posed by the contemporary COVID-19 pandemic in 2020. Depending on the duration and extent of the pandemic in Germany, the study might face several limitations including delays in recruitment, overload of participating hospitals, shortages of medical staff and thus potential confounding of the outcomes. The safety of the patients, particularly high-risk patients such as the envisaged study population, is of primary importance. Therefore, the beginning of recruitment might have to be postponed if the risk of COVID-19 is found to outweigh the anticipated benefits of the study. At this point in time, however, it is unforeseeable whether the implementation of FLS-CARE can be realised as planned. Nevertheless, several strategies are currently being developed that can be adopted depending on the course of the pandemic.

### Trial status

The present paper is the first version of the study protocol dated 26/10/2020. At the time of writing, the study is in the preparatory phase. Recruitment, which was originally planned to start in July 2020 and to be completed after 1 year, has been postponed until November 2020.

## Data Availability

Not applicable.

## Abbreviations

COVID-19: Coronavirus
CEAC: Cost-effectiveness acceptability curves
DLR: Deutsches zentrum für luft- und raumfahrt [*German aerospace centre*]
DVO: Dachverband osteologie [*osteology governing body*]
FLS: Fracture liaison services
GBA: Gemeinsamer bundesausschuss
GP: General practitioner
HIS: Hospital information system
IOF: International osteoporosis foundation
MRC: Medical research council
SHI: Statutory health insurance
QALY: Quality-adjusted life year
